# Role of the motor cortex in the generation of classically conditioned eyelid and vibrissae responses

**DOI:** 10.1038/s41598-021-96153-6

**Published:** 2021-08-17

**Authors:** Juan C. López-Ramos, José M. Delgado-García

**Affiliations:** Department of Physiology, Anatomy and Cellular Biology, Division of Neurosciences, Pablo de Olavide University, 41013 Seville, Spain

**Keywords:** Neuroscience, Physiology

## Abstract

The eyelid motor system has been used for years as an experimental model for studying the neuronal mechanisms underlying motor and cognitive learning, mainly with classical conditioning procedures. Nonetheless, it is not known yet which brain structures, or neuronal mechanisms, are responsible for the acquisition, storage, and expression of these motor responses. Here, we studied the temporal correlation between unitary activities of identified eyelid and vibrissae motor cortex neurons and the electromyographic activity of the orbicularis oculi and vibrissae muscles and magnetically recorded eyelid positions during classical conditioning of eyelid and vibrissae responses, using both delay and trace conditioning paradigms in behaving mice. We also studied the involvement of motor cortex neurons in reflexively evoked eyelid responses and the kinematics and oscillatory properties of eyelid movements evoked by motor cortex microstimulation. Results show the involvement of the motor cortex in the performance of conditioned responses elicited during the classical conditioning task. However, a timing correlation analysis showed that both electromyographic activities preceded the firing of motor cortex neurons, which must therefore be related more with the reinforcement and/or proper performance of the conditioned responses than with their acquisition and storage.

## Introduction

The classical conditioning of eyelid^1–4^ and even vibrissae (Vib)^5^ responses is a well-known experimental procedure to understand the neural processes underlying learning and memory mechanisms in mammals. This conditioning task is acquired by the paired presentation of a neutral, conditioned stimulus (CS) and by an unconditioned stimulus (US) evoking a blink or whisker response.

Several aspects of the neural centers and pathways involved in this type of associative learning are still controversial. Following the mathematical models of Marr^6^ and Albus^7^, the experimental studies of Thompson’s group^8,9^ were the first to assign to the cerebellum an essential role in classical eyeblink conditioning, whereas other authors sustained that cerebellar structures have a role in the proper performance of eyelid responses, but not in its acquisition and storage^10–13^. In relation to these seminal studies, we used timing correlation analysis aimed to test whether the cerebellar interpositus nucleus of classically conditioned mice is the origin of eyelid CRs^14^; collected results suggested that Type A interpositus neurons^12^ do not seem to be the place where CRs are generated.

Other studies propose that plastic changes underlying eyeblink conditioning are distributed across various cerebellar and extracerebellar regions, resulting in a network performance^15^. The latter hypothesis could be in accordance with those works that assign an important role in classical eyeblink conditioning to motor^16^ and prefrontal^17,18^ cortices, the hippocampus^19^, the amygdala^20^, the red nucleus^21,22^, or the claustrum^23^. As a partial agreement, delay conditioning paradigms are usually ascribed to cerebellar structures, whereas trace paradigms seem to be more related to cerebral cortical areas.

It is surprising that only a few works included the motor cortex (MC) in the relation of brain areas involved in the classical conditioning of eyelid responses^24–26^. Indeed, some past^24,25^ or recent^26^ studies have considered the putative role of this cortical structure in the acquisition of classical eyeblink conditioning in behaving mammals. In this regard, it should be remembered here the MC has a definite representation of facial muscles^27–29^, and that it is generally assumed to be a relevant brain site involved in the acquisition, storage and/or performance of acquired motor skills^30–33^. In this regard, MC dynamic activities interact with cerebellum and striatum in several types of motor learning^34–36^.

Accordingly, we have studied here the putative role of MC neurons in the classical conditioning of eyelid and Vib responses of behaving mice. As the MC area has been reported to imbricate neurons related to eyelid and Vib muscles in mice^37,38^, we have recorded, at the same time, the unitary activity of MC neurons and the electromyographic (EMG) activity of orbicularis oculi (OO) and Vib muscles during delay and trace classical conditioning paradigms. Eyelid position was determined with the help of a magnetic recording technique. We used a tone as CS, and the same air-puff as US, to stimulate eyelid and Vib simultaneously, with the aim of carrying out a timing correlation analysis^14,39^ between the two EMGs, the eyelid position, and the unitary activity of identified MC neurons. Furthermore, we have recorded the movement of the eyelid to study its kinematics and to include it in the timing correlation analysis, with the aim of revealing whether the MC area is the place where the CR is originated.

## Results

### Identification of recorded MC neurons

**Figure 1:**
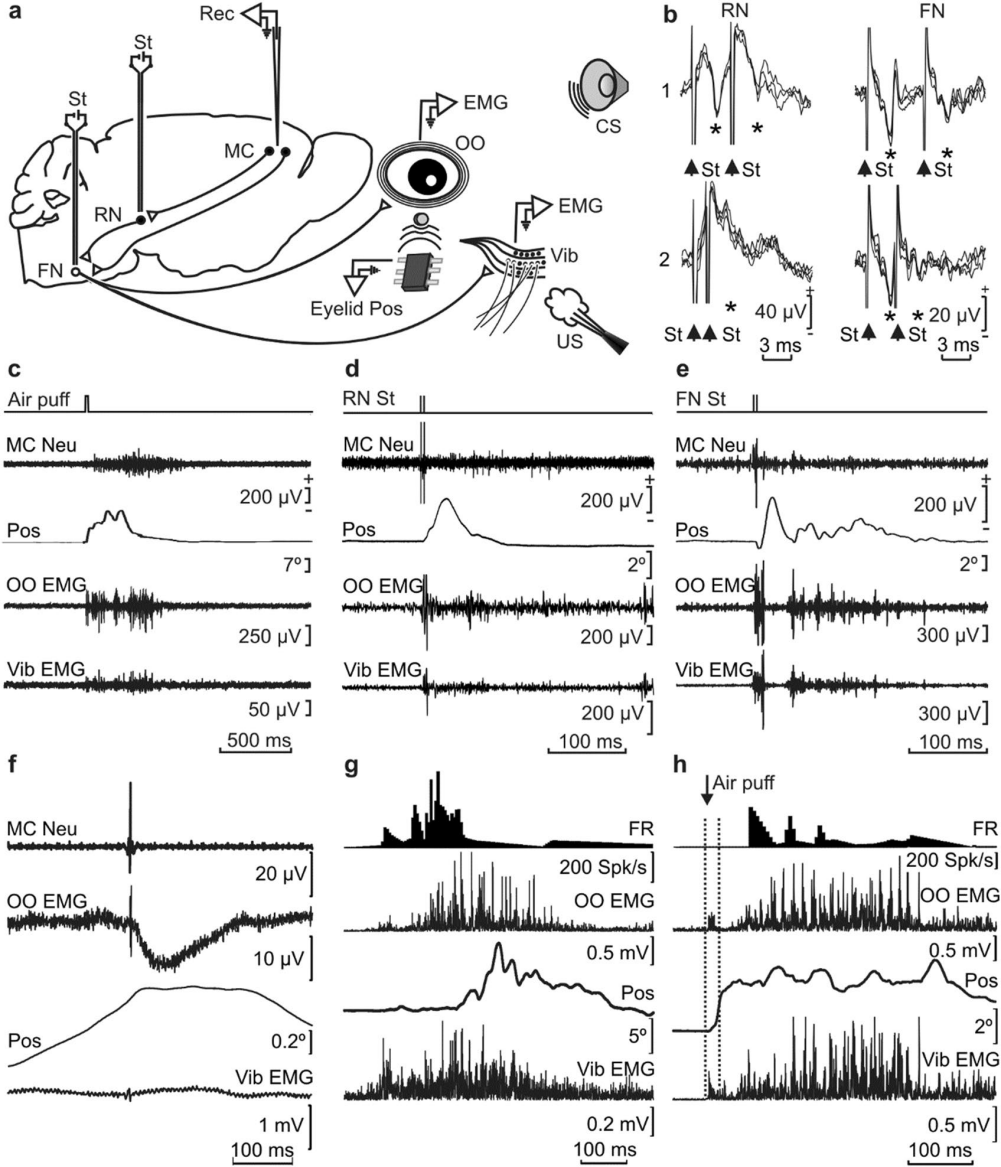
Experimental design and identification of recorded MC neurons. (**a**) Diagrammatic representation of the experimental design. Mice were chronically implanted with EMG recording electrodes in the right OO and Vib muscles. A loudspeaker was used to provide a tone as CS and an air compressor to present an air-puff to the right cornea and vibrissae as US. Eyelid position (Eyelid Pos) was determined as the voltage difference between a Hall-effect sensor located on the head-holding system and a small magnetic piece affixed to the right lower eyelid. Left MC neurons were recorded (Rec) with glass micropipettes and identified by their antidromic stimulation (St) from the left red nucleus (RN) or the right facial nucleus (FN). Ipsilateral and contralateral neurons are represented by white and black circles, respectively. (**b**) Overlapped (n = 6–8) traces of the antidromic activation (*) of representative MC neurons from the RN and FN (St) at threshold-straddling intensities and at different (RN-1, 3 ms; RN-2, 1.5 ms; FN-1, 6 ms; and FN-2, 3 ms) interstimulus intervals. Note in RN-2 and FN-2 that the antidromic activation was partially prevented. Arrows indicate stimulus artifacts. (**c**–**e**) Representative examples of the firing rate of MC neurons following the presentation of an air-puff aimed at the cornea (**c**), and of RN (**d**) and FN (**e**) stimulations, with 3 ms of interstimulus intervals. Eyelid position (Pos), OO EMG, and Vib EMG are also illustrated. (**f**) Spike-triggered activity recorded in the OO muscle (OO EMG). The triggering action potential corresponded to an identified MC neuron (MC Neu). Average was repeated 2000 times. Lower eyelid position (Pos) and EMG activity recorded in the Vib muscle are also indicated. (**g**,**h**) Typical firing rate (FR) of MC neurons recorded during the performance of a spontaneous blink (**g**) and following a strong air-puff stimulation (**h**). OO EMG, Vib EMG, and lower eyelid position (Pos) are also illustrated.

The MC recording area was approached in accordance with a mouse stereotaxic atlas^40^. As illustrated in Fig. 1b, recorded neurons were identified by their antidromic activation from their projection site—i.e., the ipsilateral red nucleus (RN) or the contralateral facial nucleus (FN)—which were chronically implanted with stimulating electrodes just in the place where their activation evoked an identifiable eyeblink. The latency of the antidromic activation of MC neurons was 2.26 ± 0.3 ms (mean ± SEM; range 1.29–3.29 ms) from the RN and 2.15 ± 0.3 ms (range 1.12–2.89 ms) from the FN. The latency for OO EMG responses was 2.73 ± 0.1 ms (range 2.25–3.3 ms) from the RN and 1.74 ± 0.1 ms (range 1.3–2.27 ms) from the FN, while the latency for Vib EMG responses was 3.02 ± 0.3 ms (range 2.1–3.76 ms) from the RN and 2.27 ± 0.2 ms (range 1.96–3.16 ms) from the FN (all these data was obtained from n = 15 measurements made in 5 animals). Examples of recorded MC neurons, OO and Vib EMGs, and lower eyelid position in response to air-puff (20 ms, 2 kg/cm^2^) and RN and FN electrical stimulations (paired pulses of 50 µs, 200 µA at 3 ms of interstimulus intervals) are illustrated in Fig. 1c–e and in Supplementary Videos 1–3. The latency of spike-triggered averaged activation of the OO muscle from MC neurons was 10.19 ± 0.6 ms (*n* = 10 measurements; range 7.6–13.9). These results further confirmed that MC neurons recorded and analyzed here project directly to the FN and have a putative disynaptic activation on the OO muscle (Fig. 1f). With respect to their firing rate, Fig. 1g,h illustrates the different patterns of MC firing profiles and timings, during a spontaneous blink, and during air-puff presentations to corneal and Vib areas.

### Evolution of conditioned responses during classical eyeblink and Vib conditioning

Before the presentation of conditioning stimuli, animals were habituated to the recording set-up, and latterly submitted to unitary recordings to collect data of MC neuronal responses to spontaneous and air-puff-evoked blinks. As an experimental rule, MC neurons were antidromically identified before the beginning of a conditioning session.

**Figure 2:**
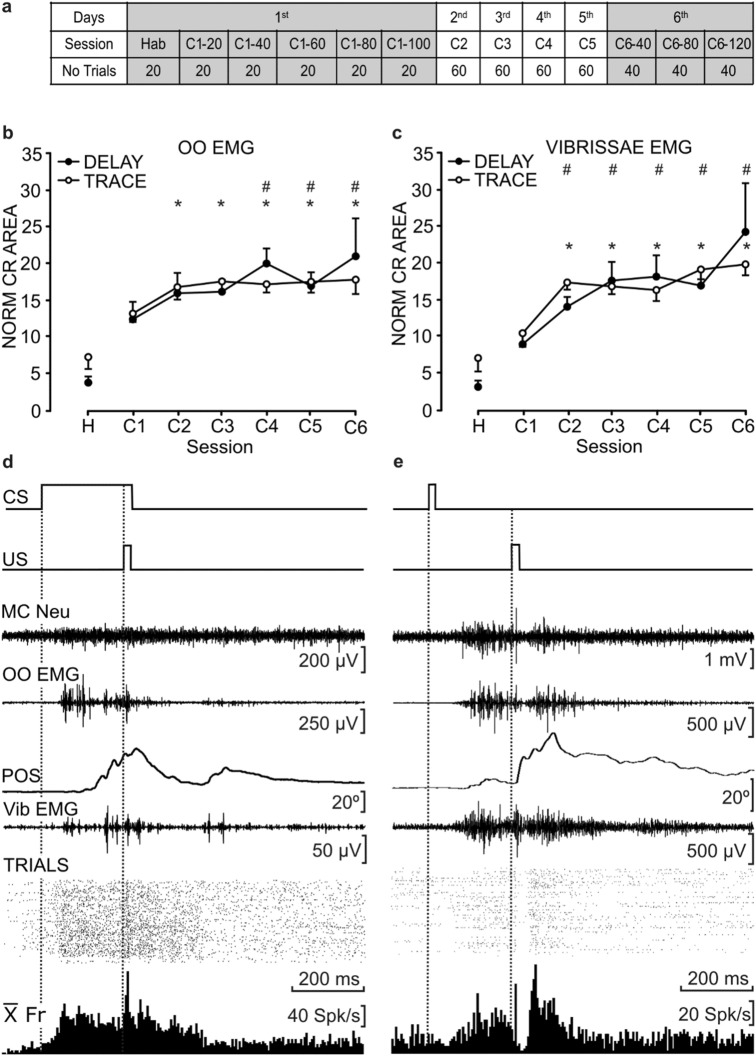
Evolution of eyeblink and Vib CRs across conditioning sessions. (**a**) Schematic representation of habituation (Hab) and conditioning (C) sessions, using both delay and trace conditioning paradigms. The 1st session consisted of 120 trials, divided in six sub-sessions of 20 trials, while the 6th (last) session consisted of 3 sub-sessions of 40 trials. Trials analyzed to obtain the learning curves (**b**,**c**) were the first 60 from each day. Shaded sessions are those from which the unitary activity of MC neurons was recorded. (**b**,**c**) Evolution of CR areas across conditioning. CR areas were computed from OO (**b**) and Vib (**c**) rectified EMG activities. One habituation (H) and six conditioning (C1–C6) sessions are represented, for both delay and trace paradigms. Data are represented as mean ± SEM of the normalized CR areas (n = 9 and 10 animals for OO and Vib, respectively, for delayed paradigms, and n = 10 and 11 animals, for OO and Vib, respectively, for trace paradigms. Symbols indicate significant differences of conditioning sessions with respect to habituation values, for delay (#) and trace (*) paradigms. *P* < 0.05, Dunnett’s post hoc one-way repeated measures ANOVA. (**d**,**e**) Representative examples of the firing activity of MC neurons recorded during delay (**d**) and trace (**e**) conditioning sessions from well-trained animals. From top to bottom are represented the conditioning paradigm (CS and US presentations), the unitary activity of the MC neuron (MC Neu), the EMG activity of the OO muscle (OO EMG), the lower eyelid position (POS), and the EMG activity of the Vib muscle (Vib EMG) for a single trial. The raster plot of > 40 successive trials (TRIALS) and its averaged firing rate (${\overline{\text{X}}}$Fr) are illustrated at the bottom.

The experimental design for the different conditioning sessions is detailed in Fig. 2a. On the first conditioning day, animals received a total of 120 trials divided in groups of 20, where the first 20 trials constituted a single habituation (Hab) session, and the other 100 consisted of conditioning (C) ones (C1–20 to C1–100). The quantitative analysis of these sessions served to determine the learning curves [H (Hab) and C1 (C1–20 to C1–60)] illustrated in Fig. 2b,c. From the second to the fifth conditioning days, animals received a 60-trial session/day, the analysis of which enabled the drawing of the learning curve corresponding to sessions C2 to C5. Finally, during the sixth day, animals received 120 conditioning trials, divided in three 40-trial sub-sessions (C6–40 to C6–100), of which the first 60 were used to draw the learning value corresponding to C6 (Fig. 2b,c). MC recordings were carried out during the first and the sixth conditioning days and served for the description of neuronal firing patterns and for the analysis of timing correlation between the different recordings, as described hereinafter.

Results showed that in habituation sessions, eyeblinks reached values of 3.75 ± 0.57 (in mV × s) in the normalized area of the CR for delay paradigm and 7.25 ± 1.09 CRs for trace conditioning paradigms, whereas Vib values reached 3.14 ± 0.58 for delay and 7.08 ± 1.25 for trace paradigms. In the sixth conditioning session, eyeblink conditioning reached values of 20.76 ± 3.5 for delay and 17.79 ± 1.33 for trace, whereas Vib conditioning reached values of 24.26 ± 4.5 for delay and 19.84 ± 1.02 for trace paradigm. Figure 2b,c illustrates the learning curves corresponding to the normalized CR areas. During eyeblink conditioning, one-way repeated measures ANOVA showed significant differences between sessions for delay [F_(6,60)_ = 2.95; *P* < 0.05] and trace [F_(6,65)_ = 4.13; *P* < 0.01] conditioning paradigms. Likewise, one-way ANOVA applied to Vib conditioning showed significant differences between sessions for delay [F_(6,48)_ = 2.9; *P* < 0.05] and trace [F_(6,73)_ = 8.15; *P* < 0.001] paradigms.

Figure 2d,e shows typical MC neurons recorded from well-trained animals during a complete (120 trials) conditioning day. The cells were activated 50–100 ms following CS presentation, and their activation almost coincided with the beginning of both OO and Vib CRs (as determined from EMG recordings). These results made necessary a further timing correlation analysis (see below) to elucidate the actual sequence of event initiations. In addition, the discharge rates of MC neurons presented different firing profiles. For example, the averaged firing rate of the neuron illustrated in Fig. 2c, corresponding to a delay conditioning paradigm, reached a peak value of ≈ 80 spikes/s at ≈ 60 ms following CS presentation, and reached a second peak after the US presentation, which allows it to be classified as a type B MC neuron (see below). In contrast, the averaged firing rate of the neuron in Fig. 2e, corresponding to a trace conditioning paradigm, increased steadily from its early activation, after the CS, until reaching a peak firing of ≈ 50 spikes/s, with a second peak of ≈ 70 spikes/s following its brief inactivation during the US presentation, allowing it to be classified as firing pattern type A (see below).

### Types of MC neuron firing pattern related to classical eyeblink and Vib conditioning

**Figure 3:**
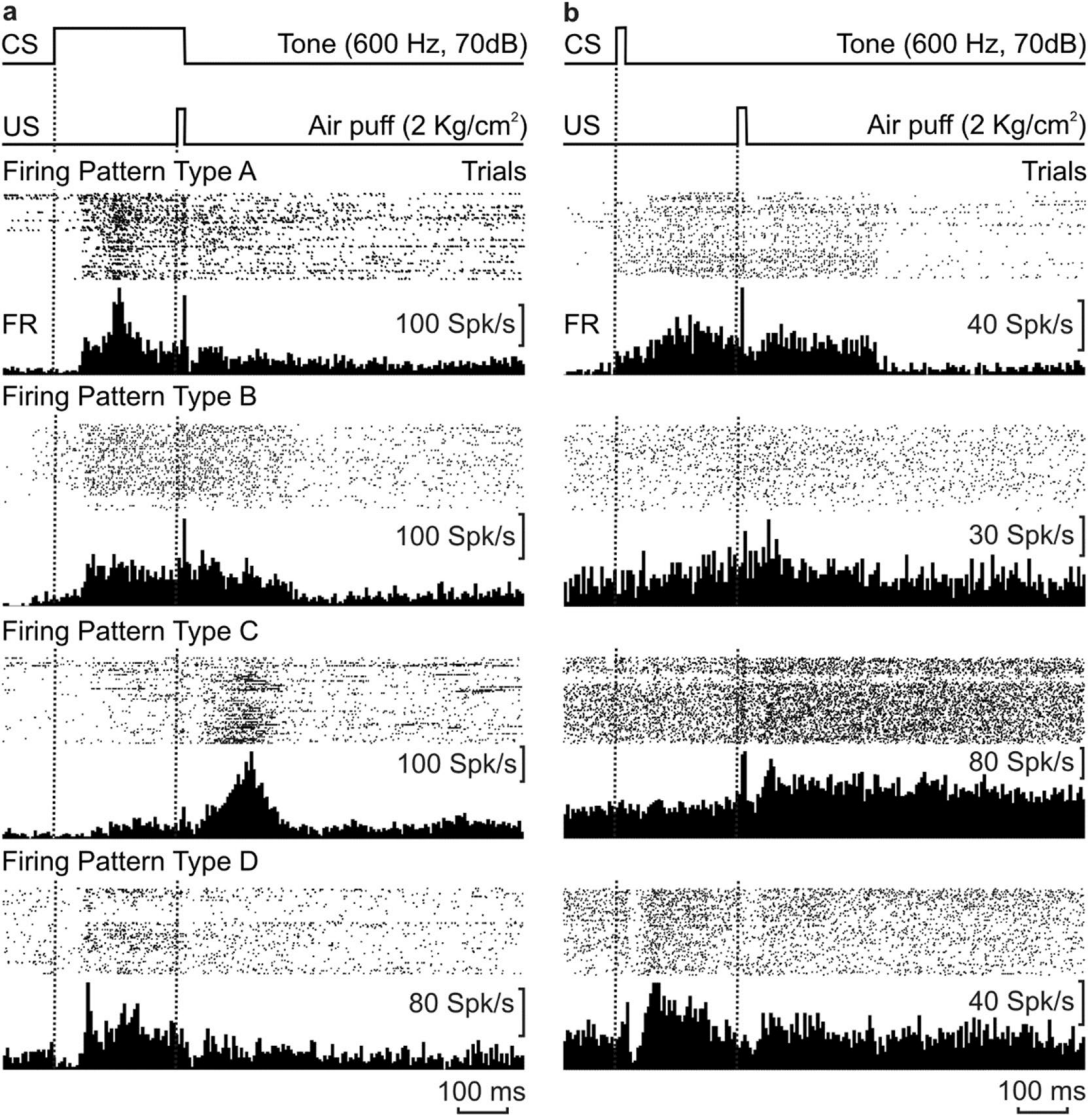
Different firing patterns of MC neurons activated during classical eyelid and Vib conditioning. (**a**,**b**) Firing patterns collected during delay (**a**) and trace (**b**) paradigms. From top to bottom are illustrated the CS–US presentations, the raster plot of spikes collected from MC neurons during a representative (n > 30) number of trials, and the averaged firing rate. Rasters and averages corresponded to the firing pattern of types A, B, C, and D of MC neurons. Type A firing pattern was characterized by an increased firing rate during the CR, and a noticeable decrease following US presentation. Type B firing presented an initial increased firing during the CR and a second increase during and after US presentation. Type C neurons presented a weak increase during the CR, reaching a maximum during and after US presentation, whereas Type D firing pattern decreased its basal rate after the beginning (delay), or brief duration (trace) of CS presentation, followed by a maximum peak, with a new decrease following the US.

Recorded MC neurons were classified in four different groups (A–D), depending on their firing rates during CR (CS–US interval) and UR (Fig. 3) periods.

The firing pattern of type A neurons (Fig. 3a,b) presented action potentials lasting > 0.5 ms, and firing rates with mean resting values ranging from 3 to 30 spikes/s. They fired a burst of action potentials during the generation of CRs, reaching a maximum at ≈130 ms from the CS, which is the principal characteristic of this pattern, with a noticeable decrease following US presentation.

Type B neurons (Fig. 3a,b) presented action potentials lasting > 0.5 ms, with a more stable firing rate than Type A pattern. This type of neuron presented two successive bursts of action potentials: one during the CS–US interval, ≈ 60 ms from the CS, more noticeable with the delay paradigm, reaching a firing of 60–120 spikes/s, and the other immediately following US presentation (30–125 spikes/s). Their firing decreased slowly after the end of the UR, reaching a spontaneous firing rate of 5–30 spikes/s thereafter.

Type C neurons (Fig. 3a,b) presented action potentials lasting > 0.5 ms, and a slow increase in their firing rates during the CS–US interval, ≈ 110 ms from CS presentation, more evident with the delay paradigm. They reached a peak discharge rate following US presentations. Like type A and B neurons, their firing decreased slowly after the end of the UR.

Finally, type D neurons (Fig. 3a,b) also presented action potentials lasting > 0.5 ms and a characteristic decrease in their basal firing rate following the beginning (delay) or brief duration (trace) of CS presentations. They reached a maximum peak (80–120 spikes/s), with a new decrease following the US presentation, and a slow decrease in firing after the end of the UR, eventually reaching basal firing rates.

### Kinetic and frequency domain properties of conditioned and unconditioned eyelid responses, compared with eyelid responses evoked by electrical train stimulation of the MC

**Figure 4:**
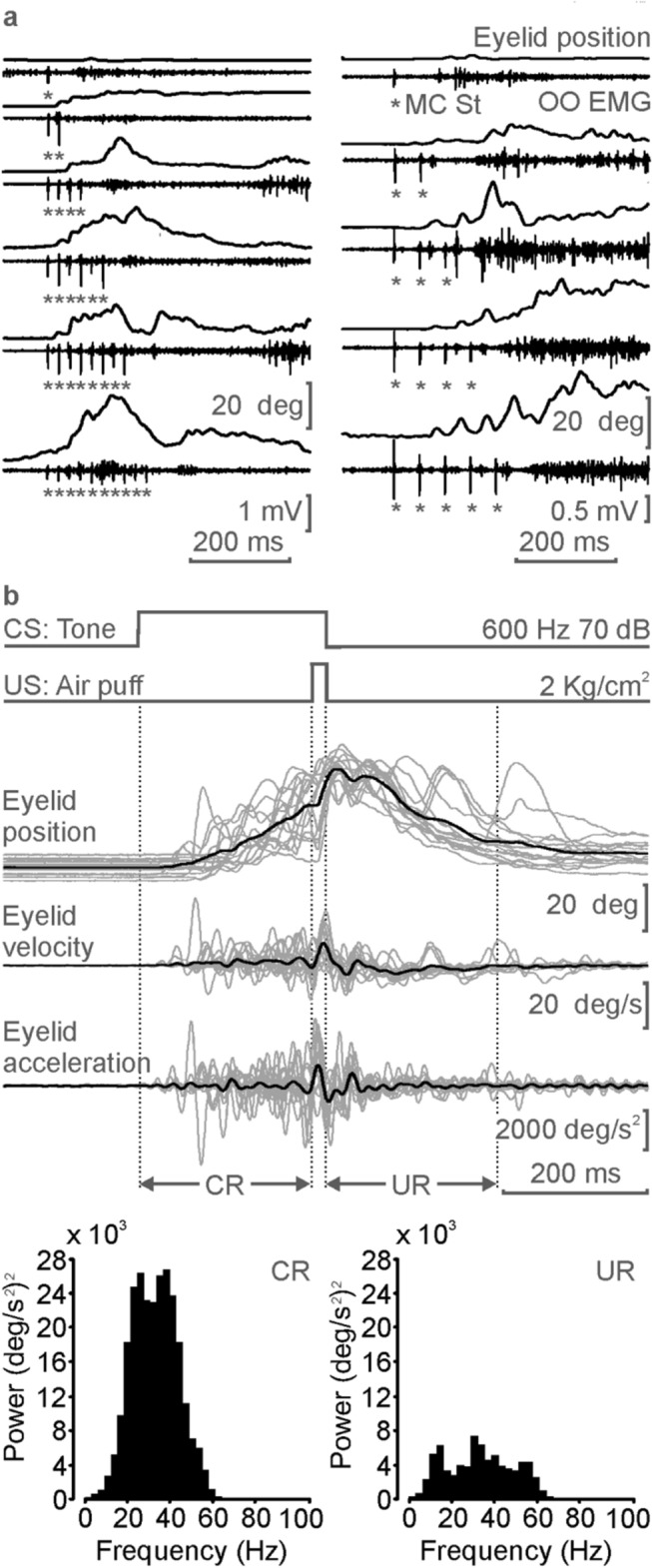
Effects of the electrical stimulation of the MC, and spectral powers of the evoked eyelid responses in conditioned mice. (**a**) Effects of the electrical stimulation of the contralateral MC on the EMG activity of the OO muscle, and on the corresponding eyelid position. The MC was stimulated with an increasing number of paired pulses (1-ms interval) at frequencies of 40 (left) and 20 (right) Hz. Each recording corresponds to an average of n ≥ 6 trials. Note the delayed response with respect to the beginning of the train stimulus, more evident with the 20 Hz pulses. (**b**) Spectral power collected from lower eyelid responses recorded with the magnetic recording technique. An average of 15 trials collected from the first conditioning session, using a delay paradigm, was differentiated once to obtain eyelid velocity and a second time for eyelid acceleration. The spectral power of CRs (bottom, left) and URs (bottom right) showed a maximum peak at frequencies ranging from 30 to 40 Hz. The 20-ms time interval between CR and UR, corresponding to the air-puff stimulation, was excluded from the study to avoid spectral artifacts due to passive eyelid movements evoked by the air pressure.

We compared the usual profiles of eyelid CRs collected with the magnetic recording technique with those evoked by train stimulation of the MC eyelid area. Trains consisted of pairs of pulses (50 µs, 200 µA) at 1 ms of interpulse interval applied at 20 and 40 Hz. As illustrated in Fig. 4a, the electrical stimulation of the contralateral MC at a frequency of 20 or 40 Hz simulates the profile and kinematics of eyelid CRs, although with a delayed response with respect to the beginning of the train of stimuli, which was longer when evoked with the 20 Hz stimulation (106 ± 6.8 ms) than with the 40 Hz one (48 ± 4.3 ms). The latency of this delayed response diminished with the repeated presentations of the trains, ranging from 206 ± 22.3 to 88.1 ± 3.2 ms from one to five pairs of pulses, for the 20 Hz trains, and from 99.2 ± 1.6 to 34.5 ± 6.2 ms from one to ten pairs of pulses, for the 40 Hz trains (see Fig. 4a and Supplementary Video 4).

Oscillatory components of the CR and UR were quantified from the power spectra of the averaged acceleration records (n = 15) of both conditioned and unconditioned responses. In the two cases, the power spectra presented a dominant peak at a frequency ranging from 30 to 40 Hz, although the power of the CR almost tripled that of the UR. This demonstrates that oscillatory properties of the eyelid are independent of the learned or reflexive origin of the movement or, in other words, that the learned character of the CR does not modify its oscillatory properties.

### Timing correlations during CRs of the classical conditioning

**Figure 5:**
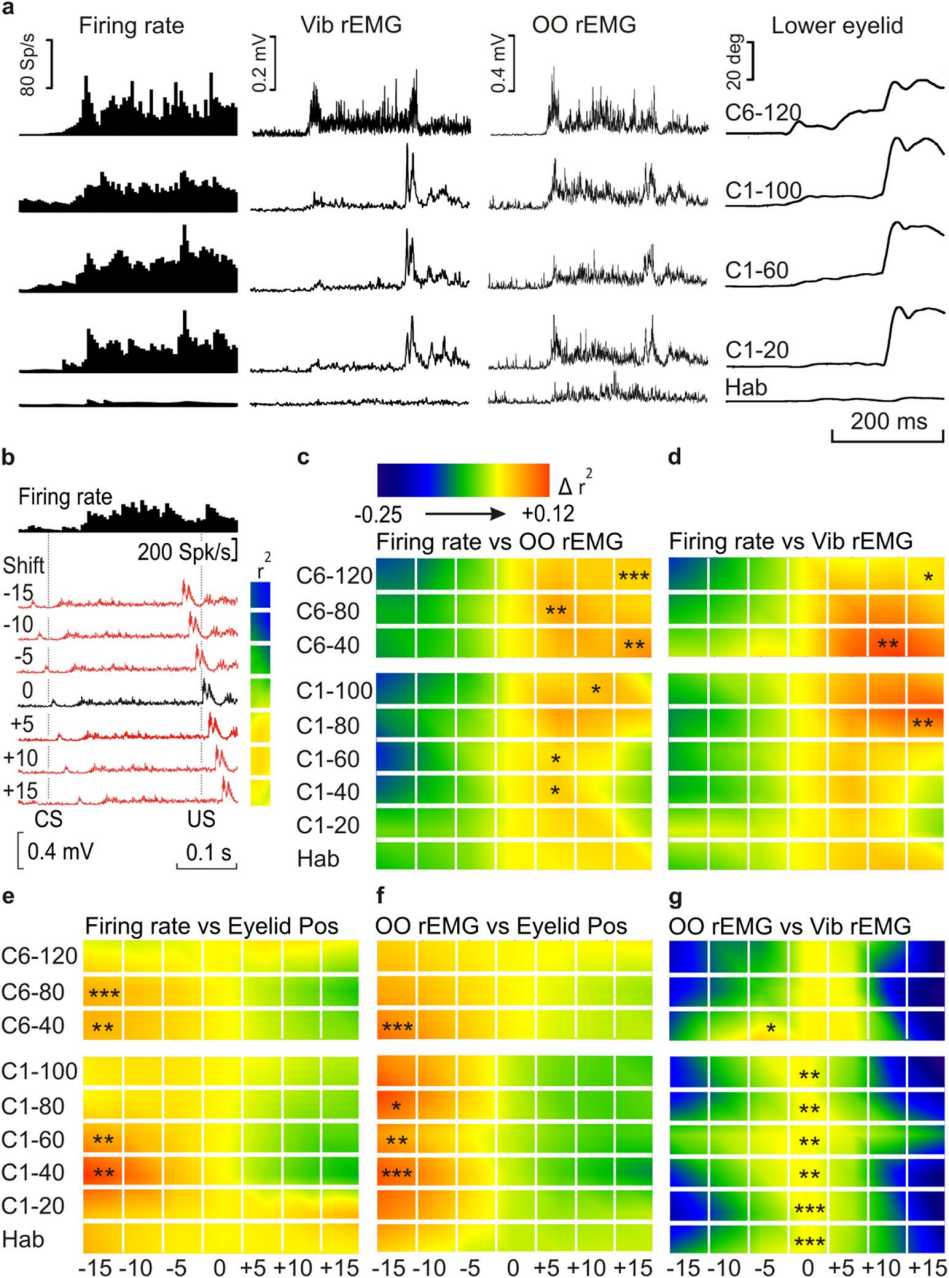
Timing correlation between MC neuron firing rate, OO rEMG, Vib rEMG, and lower-eyelid position, during eyeblink and vibrissae conditioning with a delay paradigm. (**a**) MC firing rate, Vib rEMG, OO rEMG, and lower-eyelid position corresponding to the average of 20 habituation (Hab), three groups of 20 trials from the first conditioning session (C1–20, C1–60, and C1–100), and the average of the last 40 trials from the sixth conditioning session (C6–120). (**b**) An example of the method used to compute the coefficient of determination (r^2^) between the firing rate of MC neurons and the OO rEMG. r^2^ values obtained from the comparison between a neuron firing rate and the corresponding OO rEMG, when the OO rEMG was shifted from − 15 to + 15 ms in 5-ms steps (red traces) from the actual timing (0 ms, black trace). For each comparison, the collected r^2^ value is represented by a colored square to the right of each OO rEMG recording. The calibration for color gradient values is shown at the top in (**c**). Higher r^2^ values corresponding to negative-shifted recordings indicate a delay with respect to the correlated (actual) recording, whereas higher r^2^ values corresponding to positive-shifted recordings indicate an advance with respect to the correlated one (as in the example). (**c**–**g**) Colorimetric representation of the timing correlation between MC neuron firing rate vs. OO rEMG (**c**), MC neuron firing rate vs. Vib rEMG (**d**), MC neuron firing rate vs. eyelid position (**e**), OO rEMG vs. eyelid position (**f**) and OO rEMG vs. Vib rEMG (**g**), calculated as shown in (**b**). See color code of normalized r^2^ values at the top of (**c**). Illustrated data correspond to the average of n = 7 animals for Hab and C1 and n = 6 for C6. Asterisks represent significant differences between any of the seven averaged normalized r^2^ values, corresponding to the − 15, − 10, − 5, 0, + 5, + 10, and + 15 ms shifted recordings compared, for each set of habituation and conditioning averaged group of trials, and are allocated over the higher r^2^ values, *P < 0.05; **P < 0.01; ***P < 0.001.

Following previous experimental procedures from our laboratory^14^ we carried out a timing correlation analysis to determine temporal differences between the initiation of firing of MC neurons, the OO and Vib rEMGs, and eyelid position, during the performance of CRs. For this analysis, we prepared 6 new channels copying one of each pair of the compared channels (the OO rEMG trace in the example illustrated in Fig. 5b). These new channels were shifted from − 15 to + 15 ms in 5 ms steps from the recorded (0 ms, black trace in Fig. 5b) timing. We carried out a correlation analysis between the new OO rEMG channels and the Firing rate trace (Fig. 5b) to detect which of the selected steps reached the highest coefficient of determination (r^2^). A comparison was made between regions in the CS–(US − 20 ms) interval, each one delimited by selected points detected from MC neuron firing profiles. Normalized r^2^ values were represented by colored bars corresponding to the average of training trials. The interpretation of collected results run as follows: higher r^2^ values corresponding to positive-shifted records indicate an advance of the second recording with respect to the first (correlated) one, while higher r^2^ values corresponding to negative-shifted recordings indicate a delay of the second with respect to the first. In Fig. 5b is shown a representative example corresponding to a well-trained animal. In this example, the optimal correlation was determined between the MC neuron firing profile and the + 10 ms-shifted OO rEMG channel, which means that OO rEMG events occurred ≈ 10 ms before those corresponding to the MR firing profile. In all of the cases, we used the comparison OO rEMG vs. eyelid movement (Fig. 5f) as a proper control of the analytical procedures, since muscle activity must precede eyelid movements by a fix time interval^5,14,16,22,39^.

**Figure 6:**
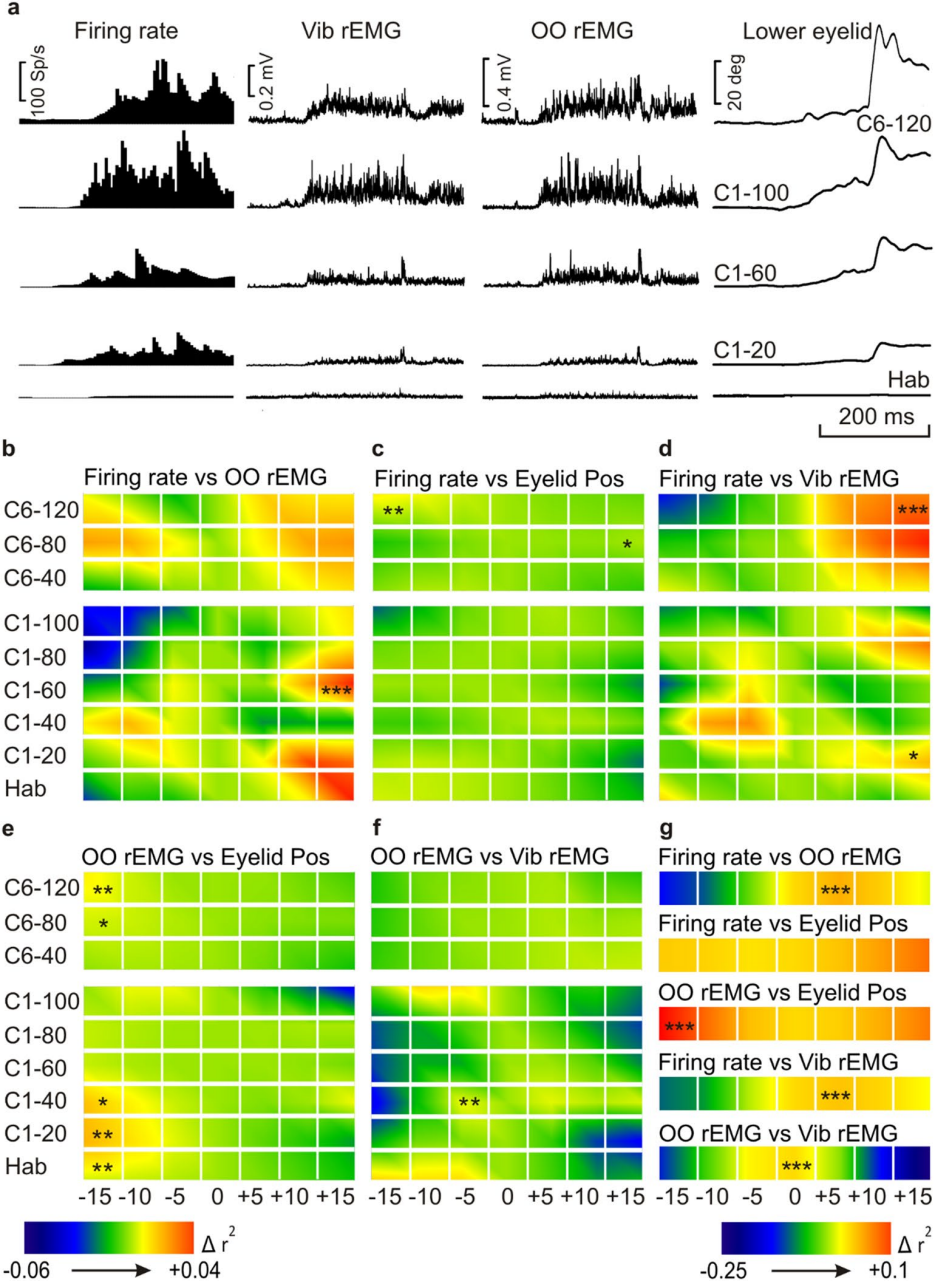
Timing correlation between MC neuron firing rate, OO rEMG, Vib rEMG, and lower-eyelid position, during eyeblink and vibrissae conditioning with a trace paradigm, and during an air-puff-evoked reflex response. (**a**) A raster representation of firing rate, Vib rEMG, OO rEMG, and lower-eyelid position corresponding to the average of 20 habituation (Hab) and three groups of 20 trials corresponding to the first conditioning session (C1–20, C1–60, and C1–100), and the average of the last 40 trials of the sixth conditioning session (C6–120). (**b**–**f**) Colorimetric representation of the timing correlation between MC neuron firing rate vs. OO rEMG (**b**), MC neuron firing rate vs. eyelid position (**c**), MC neuron firing rate vs. Vib rEMG (**d**), OO rEMG vs. eyelid position (**e**), and OO rEMG vs. Vib rEMG (**f**), calculated as shown in Fig. 5b. The color code of normalized r^2^ values is represented at the bottom left. Illustrated data correspond to the average of n = 13 animals for Hab and C1 and n = 11 for C6. Asterisks represent significant differences between any of the seven averaged normalized r^2^ values, and are allocated over the higher ones, *P < 0.05; **P < 0.01; ***P < 0.001. One way ANOVA. (**g**) Colorimetric representation of the timing correlation between MC neuron firing rate vs. OO rEMG, MC neuron firing rate vs. Vib rEMG, MC neuron firing rate vs. eyelid position, OO rEMG vs. eyelid position, and OO rEMG vs. Vib rEMG during a reflexively evoked blink. Data were calculated as shown in Fig. 5b and correspond to n = 13 animals. Asterisks represent significant differences between any of the seven averaged normalized r^2^ values, corresponding to the − 15, − 10, − 5, 0, + 5, + 10, and + 15 ms shifted recordings compared, for the averaged group of trials, and are allocated over the higher ones, *P < 0.05; **P < 0.01; ***P < 0.001.

Figures 5a and 6a show raster averages of habituation (Hab) and of representative conditioning (C1–20, C1–60, C1–100 and C6–C120) trials, corresponding to firing rate, Vib rEMG, OO rEMG, and lower-eyelid position channels of a representative animal during delay and trace paradigms, respectively. Note that the best performance of CRs took place during C6–120 conditioning trials (mostly during the delay paradigm), since the others corresponded to the first conditioning day.

### Timing correlations between neuronal firing, OO and Vib muscles activation, and eyelid position, during delay and trace conditioning paradigms

Antidromically identified MC neurons (Fig. 1b) recorded during a complete session of both delay and trace paradigms were used in this analysis. The aim was to quantify changes in the coefficient of determination (r^2^) between neuronal firing rate, OO and Vib rEMG activities, and eyelid position across the learning process. For that, mice were trained during the described paradigms, and trials were averaged in groups, as shown in Fig. 2a. MC recordings were made during the first and the sixth conditioning day and served for the description of the firing patterns (Fig. 3) and for the herein described analysis of timing correlation between the different recordings.

For timing correlations, all the recordings were compared as described above (Fig. 5b). Accordingly, trial averages of habituation (20) and conditioning (100 in groups of 20) phases of the first day, and trial averages (120 in groups of 40) of the sixth day of the experiment, corresponding to neuronal firing rate, OO and Vib rEMG activity, and eyelid movements of mice conditioned with trace and conditioned paradigms, were compared in pairs (firing rate vs. OO rEMG, firing rate vs. Vib rEMG, firing rate vs. eyelid position, OO rEMG vs. eyelid position, and OO rEMG vs. Vib rEMG). A one-way ANOVA with pairwise multiple comparison procedures was carried out to compare the normalized r^2^ values corresponding to the − 15, − 10, − 5, 0, + 5, + 10 and + 15 ms shifted recordings, and the correlated one, for each pair of compared recordings. Interestingly, whereas eyelid movements were delayed with respect to the firing (more evident in the delay paradigm) and, obviously, with respect to the OO rEMG, the OO and Vib rEMGs were advanced with respect to neuronal firing, particularly in the case of the delay paradigm, indicating that both EMG activities preceded the firing of MC neurons (see Fig. 5c–g for delay and Fig. 6b–f for trace paradigms).

The same analysis was carried out for a single averaged group of trials of one session of exposure to an air-puff-evoked reflex response (Fig. 6g). Normalized r^2^ values are represented by colored squares corresponding to each comparison made for the average of n ≥ 11 trials. Illustrated data correspond to the average of n = 13 animals. Results show that eyelid movement was delayed with respect to OO rEMG, but both OO and Vib rEMGs were advanced with respect to the MC firing. Finally, OO and Vib rEMG traces were synchronized.

In accordance with the present results, the role of MC neurons must be more related to the reinforcement and proper performance of eyelid CRs than to their initiation. Furthermore, timing correlations between firing rates and rEMGs or eyelid position indicate fine differences between paradigms.

## Discussion

Although the neural mechanisms underlying eyeblink conditioning—mostly the delay paradigm—have been delineated more completely than for any other type of mammalian learning, there are a number of critical issues that require further investigation^41^. An important current line of research gives a principal role to the cerebellum^42^, mainly for the delay paradigm, but other studies assigned that role to different brain sites, such as the motor cortex^16,26^, the hippocampus^19^, or the prefrontal cortex^43^. The acquisition of trace paradigms has been ascribed to various forebrain regions, including the hippocampus and medial prefrontal cortex, as well as the cerebellar-brainstem circuit^44^. In order to properly address these opposing hypothesis, other studies considered the kinetics and kinematic properties of some of the areas putatively involved in this learning process to accept, or reject, their putative roles in the origin of CRs^39^, or performed dynamic or timing correlation analysis to elucidate whether one specific event could be the causal origin of a following one, depending on their order of appearance^14,39^.

Here, we have addressed this question through the timing correlation analysis of the role of the MC in the origin of CRs in alert behaving mice. For that, we recorded the lower eyelid position detected with a Hall-effect sensor^45^, the OO and Vib EMGs, and the unitary activity of facial-related MC neurons. All these recordings were made at the same time, during the exposure of the animals to single air-puffs, and afterwards, during classical eyelid and Vib conditioning using delay and trace paradigms. The reason we decided to condition and record both eyelid and Vib responses is the imbricated location of their corresponding pyramidal projecting neurons in the MC area^37^.

Recorded patterns of firing neurons were classified as four different types (A–D) depending on their activation profiles. Type A firing pattern was characterized by an increased firing rate during the CR, and a noticeable decrease following US presentation. Type B firing presented an initial increased firing during the CR and a second increase during and after US presentation. Type C neurons presented a weak increase during the CR, reaching a maximum during and after US presentation, while type D firing pattern decreased its basal rate after the beginning (delay), or brief duration (trace) of CS presentation, followed by a maximum peak, with a new decrease following the US. These discharge profiles presented some coincidences with previous recordings carried out in behaving rabbits during classical eyelid conditioning^16^. Nevertheless, the short activation latency of type D neurons were not observed in rabbits, probably because this species is less sensitive to tone presentations. In any case, we preferred to talk of types of firing pattern, instead of types of neuron, because, for each recording session, it was not possible to discriminate between different types of firing neuron, which made us think that such patterns were the result of a state, rather than a casual finding of different neurons. In this regard, although at another level, previous studies of learning processes described them as functional states, rather than single functions of one or various brain areas^46^.

All of the MC neurons were activated antidromically from the FN and/or RN, although we did not detect any association between firing patterns and nucleus from which the antidromic activation was achieved. The firing of the four types of patterns started during the CS–US interval almost at the same time as the beginning of the EMGs of their corresponding CRs, which made it impossible to detect, at a glance, their order of appearance, making necessary a timing correlation analysis^14^.

We obtained learning curves, for trace and delay paradigms, corresponding to the analysis of the rectified CR areas of the recorded EMGs of both OO and Vib muscles, obtaining only a few different shapes between paradigms or between muscles, although with significant learning evolution for all. While learning curves can be obtained from different calculus, such as, for example, the percentage of CRs^47,48^ or the CR/UR ratios^14^, on this occasion the single analysis of the rectified EMG area was considered the best option, since it is an automatic process, and because a conditioned facilitation of eyeblink unconditioned responses (URs) indexed during classical eyeblink conditioning has been described^49^, which could alter the CR/UR ratios.

It has been already reported that electrical stimulation of the motor cortex in rabbits^16^ evokes eyelid profiles similar to those generated during classical eyeblink conditioning. Here, train (20 Hz and 40 Hz) stimulations of the MC reproduced, in a certain manner, the profile and kinematics of CRs recorded with the Hall-effect sensor, although with some measure of delayed response with respect to the beginning of the train stimulus. Stimulus repetitions and their increase in frequency and duration seemed to decrease this delayed initiation. This factor could facilitate the initiation of the blink, similarly to other facilitations evoked by the application of excitatory pulses, such as during the long-term potentiation^50^. It is of interest that the spectral power of both CR and UR showed a maximum peak at frequencies ranging from 30 to 40 Hz, as predicted previously from biomechanical considerations and depending on eyelid’s wight and viscoelastic properties^51–53^. Thus, it has been reported to be ≈ 10 Hz in rabbits^52^, 20 Hz in cats^2^, and 30–40 Hz in mice as already reported^53^ and confirmed here.

The small differences in maximum power frequencies and total power must be due to the fact that we excluded from the analysis the 20 ms region between CR and UR corresponding to the air-puff stimulation, with the aim of avoiding spectral artifacts that we detected, due to passive eyelid movements evoked by the air-puff pressure. Interestingly, the electrical stimulation of the motor cortex of behaving rabbits also reproduces the kinematics and oscillatory properties of eyelid CRs^16^, a result not reproduced by the electrical stimulation of cat cerebellar interpositus neurons^12^.

Timing correlation analysis between MC neurons’ firing rate, OO and Vib rEMGs and eyelid position, during both delay and trace paradigms, and during a reflexively evoked blink, showed that, whereas eyelid movements were delayed with respect to the OO rEMG, the OO and Vib rEMGs were advanced with respect to MC firing. The fact that rEMG traces preceded the initiation of neuronal firing was particularly evident for the delay paradigm (and, of course, for reflex blinks), suggesting that the CR initiations do not take place in the MC. In this regard, it is of special interest that, whereas the highest variations in r^2^ values were detected in the analysis of the delay paradigm and in the reflexed blink (see the colorimetric scales of r^2^ values in Figs. 5c–g and 6g), those variations were less than one-third as great in the trace paradigm, although significant in some cases (see the proper scales of r^2^ values in Fig. 6b–f). One possible interpretation of these results could be that the mouse MC is scarcely involved in the trace paradigm, although most recent studies have put forward the opposite view^54,55^. Another possible explanation is that the delay paradigm could be a sort of delayed response to a complex stimulus (since CS and US are overlapped) rather than a real learning process. This proposal could be supported by the fact that both delay paradigm and reflex blinks have been proposed as sharing neural pathways^56^ and described as converging in the same neural centers, such as, for example, the cerebellar interpositus nucleus^57^. In fact, a conditioned facilitation of the unconditioned reflex after classical eyeblink conditioning^49,58^ even during early stages of training^56^ has been reported to occur in delay, rather than in trace conditioning paradigms^59^.

In conclusion, results obtained here helped us to propose a positive role of MC neurons in the performance of eyelid CRs for both delay and trace conditioning paradigms^16,54,55^. Nevertheless, the timing correlation analysis showed that, at least in mice, MC neurons do not seem to be responsible for the initiation and/or the storage of those responses.

## Methods

### Experimental subjects

Experiments were carried out on C57Bl/6 adult male mice (4–6 months old; 25–30 g) obtained from an official supplier (University of Granada Animal House, Granada, Spain). They were kept on a 12:12 h light–dark cycle with constant temperature (21 ± 1 °C) and humidity (50 ± 7%). Food and water were available ad libitum. Animals were maintained in collective (up to 10) cages but were housed individually after surgery.

### Ethics statement

Experiments were carried out following the guidelines of the European Union Council (2010/276:33-79/EU), Spanish regulations (BOE 34:11370-421, 2013), and ARRIVE guidelines for the use of laboratory animals in chronic experiments. Experiments were also approved by the Ethics Committee of Pablo de Olavide University and the Junta de Andalucía, Spain (codes 06/03/2018/025 and 06/04/2020/049). We hereby confirm that all methods were performed in accordance with the above indicated guidelines and regulations.

### Surgery

Animals were anesthetized with a mixture of Ketamine (35 mg/kg) and Xylazine (2 mg/kg) i.p. All mice were implanted with pairs of electrodes in the upper OO and in the Vib muscles of the right side to record their EMG activities. Electrodes were made from Teflon-insulated, annealed stainless steel wire (50 µm in diameter, A-M Systems, Carlsborg, WA, USA) connected to a 4-pin socket (RS-Amidata, Madrid, Spain) which was affixed with dental cement to the cranial bone. Animals were also implanted with a holding system, consisting of a head-plate, fixed to the skull with the help of two small screws and dental cement, designed for their fixation to the arm of a stereotaxic apparatus. Animals were placed in a stereotaxic device as described elsewhere^14^. Then, a craniotomy was carried out over the contralateral motor cortex, and the recording chamber built around it was covered with sterile gauze and bone wax until the experimental sessions. A silver electrode (1 mm in diameter), in contact with the dura mater, was attached to the left parietal bone as ground. A 1-mm diameter hole was made in the skull, over the underlying contralateral red nucleus (AP, − 3.52; L, − 0.62; and D, 3.7) and the underlying ipsilateral facial nucleus (AP, − 6; L, − 1.25; and D, 5.75)^40^, and covered with bone wax. In a second surgical step, behaving mice were fixed by their head-plate to the holding system, and implanted, using the hole previously made, in the red nucleus or in the facial nucleus, with a pair of stimulating electrodes made from 50 µm, Teflon-coated tungsten wire (Advent Research Materials Ltd, Eynsham, England), soldered to a 2-pin socket and fixed to the skull with acrylic cement. The final position of these stimulating electrodes was determined by eyelid-closing movements evoked by pairs of pulses (1-ms interpulse interval) applied in the selected nucleus (Fig. 1a).

### Classical conditioning

Animals were fixed individually by their head-plate to the bars of a stereotaxic apparatus, while their legs rested on a muffled running wheel adapted to the animals’ size and habituated to it during a few sessions. The recording room was softly illuminated during the experiments and provided with a 50-dB background white noise. Animals were trained using both delay and trace conditioning paradigms. For this, animals were presented with a tone (2400 Hz and 85 dB, lasting 250 ms for delay and 20 ms for trace) as CS, followed 250 ms later by an air-puff (20 ms, 2 kg/cm^2^) as US. CS–US presentations were separated at random by 30 ± 5 s. For habituation sessions, only the CS was presented. During the first and the last days of training, identified MC neurons were recorded to evaluate timing correlation between their activity, the OO and Vib EMGs, and the eyelid position. Distribution of habituation and conditioning sessions is schematized in Fig. 2a.

We recorded the EMG activity of the OO and Vib muscles through differential amplifiers within a bandwidth of 1 Hz to 10 kHz (Grass Technologies, West Warwick, USA). Recorded traces were stored online on a computer with the help of an analog/digital converter (CED 1401 Plus, Cambridge Electronic Design, Cambridge, England), at a sampling frequency of 11–22 kHz and with an amplitude resolution of 12 bits. Data collected from the first 60 trials during the six training days were analyzed off-line. CR areas were computed from collected EMG recordings with the Signal software, version 5.11 (Cambridge Electronic Design Ltd. http://ced.co.uk/). For this, the rectified and filtered (250 Hz, high pass) EMG area collected from the CS–US interval (i.e., 250 ms) was quantified (in mV × s). The 20-ms interval during the US artifact was excluded from the analysis.

Statistical analyses were carried out using the Sigma Plot software, version 11.0 (Systat Software, Inc. https://systatsoftware.com/), for a significance level of *P* < 0.05. Mean values are followed by their SEM. A one-way repeated measures ANOVA was calculated, and a Dunnett’s post hoc method was performed.

### Recording and stimulating procedures

We recorded the unitary activity of antidromically identified MC neurons during classical conditioning of eyelid and Vib responses. Neuronal unitary activity was recorded with the help of an AM 3000 AC/DC differential amplifier (A-M Systems, Inc., Carlsborg, WA, USA). Unitary recordings were performed with glass micropipettes filled with 3 M NaCl (2–5 MΩ of resistance) coupled to a preamplifier and filtered in a bandwidth of 1 Hz to 25 kHz. Antidromic field potentials were evoked by electrical stimulation (paired, cathodic) 500 µs, < 0.6 mA, 500-µs to 10-ms of interpulse interval) of the ipsilateral red nucleus or the contralateral facial nucleus, programmed with a CS-220 stimulator across an ISU-220 isolation unit (Cibertec, Madrid, Spain). Criteria were systematically followed to determine whether the recorded and the activated neurons were the same^60,61^. At the end of each session, the recording micropipette was removed, and the recording chamber sterilized and closed with bone wax. Eyelid movements were recorded as the voltage difference between a Hall-effect sensor and a magnetic cylinder (1.2 mm Ø, 0.5 mm height) fixed to the lower eyelid. Maximum angular displacements of the lower eyelid were ≈ 30° for all the animals. For the sake of homogeneity, the gain of the recording system was adjusted to yield 1 V per 10°.

Before experiment onset, air-puffs were applied to the eyes and Vib, to record eyeblink and Vib movement-related MC neurons, for further analysis of timing correlation between MC neurons’ firing rate, OO and Vib EMG, and eyelid position.

After conditioning experiments, some animals were again affixed to the stereotaxic apparatus and electrically stimulated in the contralateral MC with tungsten wires. Stimulation consisted of an increasing number of paired (cathodal, square, 50-µs, < 500-µA, 1-ms interval) pulses, at frequencies of 40 and 20 Hz, programmed with a CS-220 stimulator across an ISU-220 isolation unit (Cibertec, Spain). OO EMG and eyelid position were recorded for further analysis.

Selected eyelid responses to the different stimuli used here were recorded with a fast charge-coupled device (CCD) camera (Pike F-032, Allied Technologies, Stadtroda, Germany) to determine the profile of the different types of evoked blinks (Supplementary Videos 1–4).

At the end of the experiments, we carried out a histological study to determine the proper location of stimulating and recording sites, following routine procedures of our laboratory^14,26^.

### Data collection, storage and analysis

The unitary activity of identified MC neurons, as well as the unrectified EMG activity of the OO and Vib muscles, the lower-eyelid position, and 1-V rectangular pulses corresponding to CS, US, and electrical stimuli presented during the different experiments, were stored digitally on a computer through an analog–digital converter (1401-Plus, Cambridge Electronic Design) for quantitative off-line analysis^12^. Collected data were sampled at 25 kHz for unitary recordings or at 10 kHz for EMGs and eyelid-position recordings, with an amplitude resolution of 12 bits. A computer program (Spike2, version 7.18, Cambridge Electronic Design Ltd. http://ced.co.uk/) was used to display single and overlapping representations of unitary activity, EMG activity of the OO and Vib muscles, and eyelid position.

As described in detail in previous studies from our laboratoy^51,52^, velocity and acceleration traces were computed digitally as the first and second derivative of eyelid-position records, following a low-pass filtering of the collected data (cut-off at 50 Hz). The power spectra of eyelid movements were calculated from the corresponding acceleration profiles. The power spectrum (i.e., power of the spectral density function) was calculated using a fast Fourier transform to determine the relative strength of the different frequencies present in eyelid responses^12,51,52^.

### Correlation analysis

For events average and the analysis of putative correlations between neuronal firing rates, rEMG profiles, and eyelid positions, all data collected with the Spike2 program, version 7.18, were exported to the Signal software, version 5.11. Next, as described before^14^, new channels, copying the selected rEMGs and eyelid position files, were created and shifted − 15 ms, − 10 ms, − 5 ms, + 5 ms, + 10 ms, and + 15 ms from the original timing, and, with the help of two active cursors, segments of the CS–US interval were delimited by turning points automatically detected on the firing-rate channel, and the area of each of them was calculated (Fig. 5b) and stored. Then, and with the help of Sigma Plot software, version 11.0, we carried out a global analysis of linear correlations between those MC-neuron firing-rate segment areas, and the segment areas corresponding to the original and the shifted rEMG channels. The same procedure was used to calculate the coefficients of determination between neuronal firing rates and lower-eyelid positions, between OO rEMG profiles and lower-eyelid positions, and between the two rEMGs profiles. A 20-ms interval, corresponding to US artifacts of original and shifted channels, was excluded from the analysis.

For the analysis of the first and sixth conditioning sessions, the coefficient of determination (r^2^) was calculated from averages of groups (20 habituation and 5 groups of 20 conditioning events, for the first, and 3 groups of 40 events for the sixth), and the r^2^ values of n ≥ 6 animals were normalized and averaged to obtain the final normalized r^2^, which was represented by colorimetric gradients. A single air-puff-evoked blink session was also analyzed.

## Supplementary Information

Supplementary Captions.

Supplementary Video 1.

Supplementary Video 2.

Supplementary Video 3.

Supplementary Video 4.

## Abbreviations

ANOVA: Analysis of variance
C: Conditioning
CR: Conditioned response
CS: Conditioned stimulus
EMG: Electromyography
FN: Facial nucleus
Hab: Habituation
MC: Motor cortex
OO: Orbicularis oculi
rEMG: Rectified EMG
RN: Red nucleus
SEM: Standard error of the mean
UR: Unconditioned response
US: Unconditioned stimulus
Vib: Vibrissae
