# Supplementary material for: Role of the motor cortex in the generation of classically conditioned eyelid and vibrissae responses
Source: Sci Rep. 2021 Aug 17;11:16701. doi: 10.1038/s41598-021-96153-6 (PMC8371024; doi:10.1038/s41598-021-96153-6)
Supplement: Supplementary file 1 — Supplementary Captions. [file 41598_2021_96153_MOESM1_ESM.docx]

**Supplementary Information**

**Role of the motor cortex in the generation of classically conditioned eyelid and vibrissae responses**

**Juan C. López-Ramos^*^, José M. Delgado-García**

**Supplementary Video legends**

**Supplementary Video 1.** Eyelid response evoked by the presentation of an air-puff (20 ms, 2 kg/cm^2^) to the right cornea.

**Supplementary Video 2.** Eyelid response evoked by the electrical stimulation of the contralateral red nucleus (paired pulses of 50 µs and 200 µA presented at 3 ms of interpulse intervals).

**Supplementary Video 3.** Eyelid response evoked by the electrical stimulation of the ipsilateral facial nucleus (paired pulses of 50 µs and 200 µA presented at 3 ms of interpulse intervals).

**Supplementary Video 4.** Eyelid response evoked by the electrical train stimulation of the contralateral motor cortex. Trains consisted of pair of pulses of 50 µs and 200 µA (1-ms of interval) applied at 40 Hz.
